# Management of Late-Onset Complete Atrioventricular Block Post Transcatheter Closure of Perimembranous Ventricular Septal Defects

**DOI:** 10.3389/fped.2019.00545

**Published:** 2020-01-21

**Authors:** Lijian Xie, Han Zhang, Rufang Zhang, Tingting Xiao

**Affiliations:** ^1^Department of Cardiovascular, Shanghai Children's Hospital, Shanghai Jiaotong University, Shanghai, China; ^2^Department of Cardiothoracic Surgery, Shanghai Children's Hospital, Shanghai Jiaotong University, Shanghai, China

**Keywords:** complete atrioventricular block, perimembranous ventricular septal defect, transcatheter, surgical, occluder

## Abstract

Long-term late-onset complete atrioventricular block (CAVB) is one of the most serious complications of transcatheter closure of perimembranous ventricular septal defect (pmVSD); it can cause an Adams-Stoke attack or even sudden death. Transcatheter closure of pmVSD is not approved by the FDA, yet the procedure has proved to be a successful alternative to a surgical strategy in China. Although transcatheter closure of pmVSD is widely and successfully performed, especially in China, late-onset CAVB is still difficult to avoid. Here, we report a case with late-onset CAVB post transcatheter closure that was successfully treated. By doing so, we reassess the safety of pmVSD occluder closure and highlight that use of this procedure should adhere to more stringent indications.

## Introduction

Although transcatheter closure of perimembranous ventricular septal defect (pmVSD) is not approved by the FDA (full text available at https://www.accessdata.fda.gov/cdrh_docs/pdf4/p040040b.pdf), the procedure has proved a successful alternative to a surgical strategy in China. The incidence of complications has been decreased significantly by selecting suitable patients and using a modified VSD occluder for transcatheter closure. However, complete atrioventricular block (CAVB), especially late-onset CAVB, is one of the most serious complications and is difficult to prevent. Management of late-onset CAVB post transcatheter closure of pmVSD is controversial. Few studies on the management of late-onset CAVB have yet been reported (1–3). Here, we report a case of late-onset CAVB post transcatheter closure that was successfully treated. It is important to evaluate the safety of the procedure in younger children through this case report.

## Case Report

**Figure 1:**
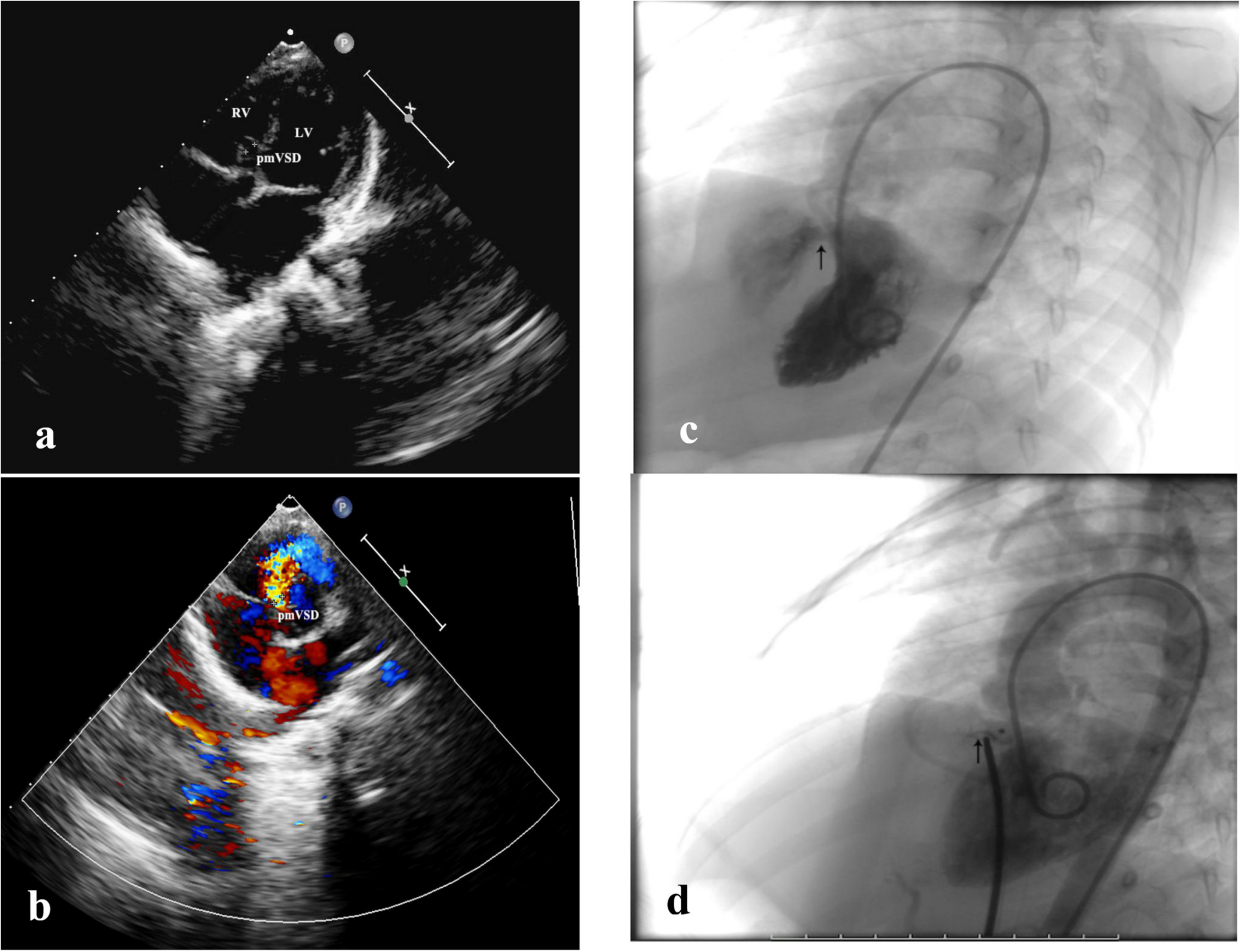
Echocardiography and DSA imaging. **(a)** Two-dimensional echocardiography showing pmVSD in the apical five-chambered view. **(b)** Color Doppler echocardiography showing pmVSD (left to right shunt) in the short-axis view. **(c)** DSA image showing left to right shunt through the pmVSD in the left anterior oblique position. **(d)** DSA image showing no residual shunt post transcatheter closure with MODO.

**Figure 2:**
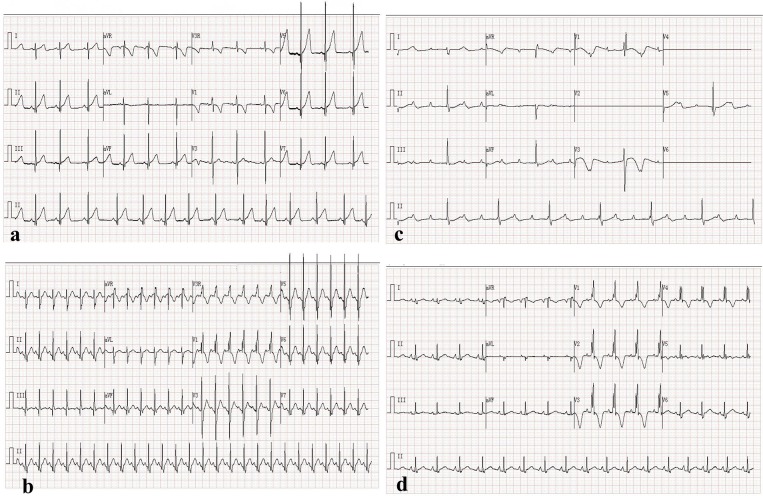
Electrocardiogram. **(a)** Normal sinus rhythm pre occluder closure. **(b)** Normal sinus rhythm and CRBBB immediately post closure. **(c)** CAVB 2.5 years post closure. **(d)** Normal sinus rhythm recovery and CRBBB post surgical removal of occluder 3 months later.

A 27-month-old male (body weight 12 kg and height 94 cm) was diagnosed as having pmVSD. The patient had a history of recurrent respiratory infections. III/6 systolic murmur was detected by precordial auscultation. Transthoracic echocardiography (TTE) revealed the diameter of the pmVSD to be 4 mm (Figure 1) and the left ventricle diastolic diameter to be 3.1 cm (z-score of 0.04). A 12-lead electrocardiogram (EKG) and 24-h electrocardiographic Holter showed normal sinus rhythm (Figure 2). Transcatheter closure of the pmVSD was successfully performed. The 6-mm symmetric ventricular septal device was a modified double-disc VSD occluder (MDVO) (Shanghai Shape Memory Alloy) (Figure 1). The position of the device was checked and found to be satisfactory, and no residual shunt was found by TTE. EKG showed normal sinus rhythm and complete right bundle branch block (CRBBB) immediately post pmVSD closure (Figure 2).

Within 2-years follow-up, no clinical discomfort was complained of. On the other hand, there was no significant body weight gain compared with that pre-transcatheter closure. TTE revealed no displacement of the occlude, and EKG showed normal sinus rhythm and CRBBB post closure (Figure 2). Regrettably, 24-h electrocardiographic Holter was not taken during the 2 years after pmVSD closure. Two and half years post pmVSD closure, astonishingly, a routine follow-up examination showed a high degree atrioventricular block, and CAVB (Figure 2); the patient's parents refused to implant a permanent pacemaker. Three months after that, the patient was admitted to our hospital due to a short Adams-Stokes attack (>1 min) and a temporary pacemaker was implanted immediately. Fulminant myocarditis was ruled out because the myocardial enzyme was normal. CK-MB was 35 U/L, and LDH was 214 U/L. Myocardial dysfunction caused by CAVB was confirmed because the N-BNP was very high (4007.73 pg/ml).

Because the CAVB happened two and half years post transcatheter closure and the level of myocardial enzyme was also normal, we thought that steroid treatment may not be helpful. The routine treatment is to implant a permanent pacemaker. However, the patient's parents disagreed with this plan. We decided to surgically remove the occluder and repair the pmVSD after further discussion. Implantation of a permanent pacemaker would have been our next approach in case of non-recovery of the function of the atrioventricular node (AV node). The routine pmVSD repair surgery was performed. The occluder was observed under the tricuspid septum valve via right atrial incision and it was removed carefully. The diameter of the pmVSD via the right ventricular surface was 8 mm, and it was closed by continuous suture with a self-pericardial patch with epicardial pacing performed.

The patient's EKG showed CAVB post surgical management for 2 days, and epicardial pacing was performed. It then gradually shifted to second degree atrioventricular block (2:1 type). One week later, sinus rhythm recovered, and CRBBB still existed (Figure 2). During 6 months of follow-up, EKG showed sinus rhythm and CRBBB, and no atrioventricular block occurred according to Holter monitoring.

## Discussion

CAVB is one of the most serious complications of transcatheter closure of pmVSD. The incidence of CAVB associated with transcatheter closure of pmVSD has been shown to be approximately 1~6%, depending on different clinical experience and occluder selection (4). Recently, a meta-analysis of transcatheter device closure of pmVSD revealed that the incidence of CAVB was 1.1% (5). CAVB can occur during any period, and it cannot currently be predicted (6–8). The possible mechanism of CAVB after transcatheter closure may be related to inflammatory edema of membranous interventricular septum tissues. The septum tissues near the AV node and conduction branches are compressed by the memory alloy of the VSD occluder (9).

The first successful closure of pmVSD with MDVO was reported in 2002 in China (10). Compared with the AMPLATZER device, the longer waist of the MDVO causes less strain to be placed on the membranous interventricular septum, so it can theoretically reduce the risk of CAVB (11). It is difficult to compare the advantages and disadvantages of transcatheter device closure and established surgery since follow-up studies of patients who underwent transcatheter device closure are limited to no more than 20 years. In contrast, the surgical approaches can be traced back 60 years. A meta-analysis of transcatheter device closure of pmVSD showed the incidence of CAVB to be 1.1% (5). No statistical difference is found when comparing this with the CAVB rate (<1%) of surgical VSD repair (12).

Late-onset CAVB after transcatheter closure of pmVSD is a serious and intractable complication. Steroid treatment has proved its efficacy in the immediate onset of CAVB post occluder closure. The recovery of sinus rhythm via steroid therapy in patients with late-onset CAVB has been found to be very rare (13). It has never been reported that late-onset CAVB recovered from steroid treatment. Implantation of a permanent pacemaker is a major management approach in patients with late-onset CAVB (1–3). The surgical removal of the occluder together with surgical repair of pmVSD was reported in a patient with early-onset CAVB (3). Longtime late-onset (two and half years post transcatheter closure) CAVB post transcatheter closure has rarely been reported. Furthermore, successful surgical management of such cases has never been reported until now. Therefore, the experience of our case has given us the following insights. Firstly, transcatheter closure of pmVSD has been widely and successfully performed in China; it is still vital to strictly abide by the indications of this procedure. The international inclusion and exclusion criteria of transcatheter closure of pmVSD were referenced by Fu et al. (14) and Zhou et al. (4). Retrospectively, the patient was slightly underweight (12 kg) when he underwent transcatheter closure. We should be more conservative with such young patients, even though the body weight was suitable for IIa type indication of occluder closure in China (15). According to the z-value evaluation, the LV of the patient was not enlarged. In fact, it was not an appropriate indication for pmVSD occluder closure. This is a serious lesson for us. Secondly, if CRBBB occurs immediately post occluder closure, we need to be alert that it may develop into high degree AVB or CAVB, even at a very low incidence. However, there is no literature to support this viewpoint. As a result, whether to withdraw the occluder when CRBBB occurs in the procedure is worth further discussion. If we locate the occluder in the aneurysm, the compression of the membranous interventricular septum may be avoided. However, a device located in the aneurysm will cause an increase in the anterior flow velocity across the tricuspid valve. Last but not least, through our successful surgical management of this late-onset CAVB, decompression of the AV node by removing the occluder played an important role in the recovery of sinus rhythm. Compared with implantation of a pacemaker, surgical removal of the occluder is more beneficial for the patient's quality of life. In a word, surgical removal of the occluder is an alternative treatment for late-onset CAVB.

## Conclusion

Although transcatheter closure of pmVSD is widely and successfully performed, especially in China, late-onset CAVB is still difficult to avoid. It is very important to evaluate the safety of this procedure. Use of this procedure should adhere to more conservative indications, especially in younger patients.

## Data Availability Statement

All datasets generated for this study are included in the article.

## Ethics Statement

The studies involving human participants were reviewed and approved by The Institutional Review Board of Shanghai Children's Hospital. Written informed consent was obtained from the patient's mother (legal guardian) for the publication of this case report.

## Author Contributions

LX, TX, and RZ designed and operated the project. HZ provided clinical data. LX and HZ wrote the manuscript. All authors read and approved the final manuscript.

### Conflict of Interest

The authors declare that the research was conducted in the absence of any commercial or financial relationships that could be construed as a potential conflict of interest.
